# Orbital lymphatic vessels: immunohistochemical detection in the lacrimal gland, optic nerve, fat tissue, and extrinsic oculomotor muscles

**DOI:** 10.5935/0004-2749.20210035

**Published:** 2021

**Authors:** Renato Wendell Ferreira Damasceno, Juliana Arôxa Pereira Barbosa, Lucas Rafael Costa Cortez, Rubens Belfort Jr.

**Affiliations:** 1 Universidade Estadual de Ciências da Saúde de Alagoas, Maceió, AL, Brazil; 2 Hospital Universitário Professor Alberto Antunes, Universidade Federal de Alagoas, Maceió, AL, Brazil; 3 Department of Ophthalmology and Visual Sciences, Escola Paulista de Medicina, Universidade Federal de São Paulo, São Paulo, SP, Brazil

**Keywords:** Lymphatic vessels, Orbit, Optic nerve, Lacrimal apparatus, Oculomotor muscles, Adipose tissue, Microscopy, Vasos linfáticos, Órbita, Nervo óptico, Aparelho lacrimal, Músculos oculomotores, Tecido adiposo, Microscopia

## Abstract

**Purpose:**

To identify the lymphatic vessels in orbital specimens from human cadavers using light microscopy and immunohistochemical analysis.

**Methods:**

A postmortem study included 10 orbital specimens from 10 human cadavers. The orbital specimens were obtained no later than 12 hours after death. The orbital specimens were dissected into lacrimal gland, optic nerve, fat tissue, and oculomotor muscles. The histologic criteria to qualify as a lymphatic vessel were thin-walled channels of endothelium without a well-developed basal membrane and with an erythrocyte-free, irregular lumen. The immunohistochemical criteria were irregularly shaped, thin-walled vessels with an erythrocyte-free, irregular lumen and immunopositivity for podoplanin D2-40.

**Results:**

The lacrimal gland, optic nerve, fat tissue, and extraocular muscle sections were positively stained with podoplanin D2-40.

**Conclusions:**

This study demonstrated lymphatic vessels in the human orbit, more precisely, in the lacrimal gland, dura mater of the optic nerve, adipose tissue, and extrinsic oculomotor muscles via light microscopy and immunohistochemistry.

## Abstract (pt)

**Objetivos:**

Identificar vasos linfáticos em espécimes orbitários de cadáveres humanos através de microscopia óptica e análise imunohistoquímica.

**Métodos:**

Um estudo *postmortem* incluiu dez espécimes orbitários provenientes de dez cadáveres humanos. Todos os espécimes orbitários foram obtidos até 12 horas após a morte com uma técnica cirúrgica de exenteração orbitária e dissecados em glândula lacrimal, nervo óptico, gordura órbitária e músculos extraoculares. Para classificar como um vaso linfático, os critérios histológicos incluíram vasos endoteliais de parede única sem membrana basal bem desenvolvida, irregulares e lúmen sem hemácias, e os critérios imunohistoquímicos incluíram vasos endoteliais de parede única, com formato irregular e lúmen sem hemácias e reagentes a podoplanina D2-40.

**Resultados:**

As lâminas histológicas de glândula lacrimal, nervo óptico, tecido adiposo e músculos extraoculares reagiram positivamente a podoplanina D2-40.

**Conclusão:**

Este estudo demonstrou vasos linfáticos na órbita humana, mais exatamente, na glândula lacrimal, no nervo óptico, na gordura orbitária e nos músculos extrínsecos extraoculares via microscopia óptica e imunohistoquímica.

## INTRODUCTION

The lymphatic system is essential for maintaining the normal balance of body fluids^(1,2)^. This system continually transports interstitial macromolecules from the extracellular space into the venous circulation^(2)^. Additionally, the lymphatic system is responsible for fat absorption and transports immune cells to the lymph nodes, thus playing a prime role in immunity^(2)^. Although the organization of the lymphatic system is parallel to that of the blood system, the lymphatic vessels are not uniformly distributed throughout the body in a similar way^(1)^. Whereas the ocular adnexa (conjunctiva, eyelids, and lacrimal drainage system) are rich in blood and lymphatic vessels, some well-vascularized tissues, such as the central nervous system, have no lymphatics^(1)^.

The human orbit, an ocular adnexa richly vascularized with blood vessels, is a space bounded by rigid bones and fascial sheaths^(1,2)^. The orbital blood capillaries are unfenestrated and carry proteins into the extravascular space continuously; however, these proteins are not readily reabsorbed owing to the concentration gradient^(1,2)^. Thus, this protein-rich interstitial fluid must leave the orbit and be returned to the vascular compartment to avoid an increase in the intraorbital pressure.

Several studies have shown that lymphatic vessels may play an important role in various disorders^(3-15)^. A better understanding of the orbital lymphatic system may significantly increase our knowledge of immu neasso ciated orbital diseases, such as orbital inflammatory diseases and Graves’ ophthalmopathy, and the spread of malignant neoplasms, including carcinoma, lymphoma, and melanoma.

The purpose of this study was to determine whether lymphatic vessels are present in orbital specimens from human cadavers, with special regard to the lacrimal gland, optic nerve, fat tissue, and extraocular muscles, using light microscopy and immunohistochemical analysis.

## METHODS

### General information

This postmortem study was performed on 10 orbital specimens from 10 human cadavers between January 1 and December 31, 2016. The orbital specimens were obtained no later than 12 hours after death using a modified surgical technique of orbital exenteration at the Autopsy Service, Universidade Estadual de Ciências da Saúde de Alagoas, Maceió, Brazil. The orbital specimens were dissected into lacrimal gland (palpebral and orbital lobes), optic nerve, fat tissue (lower inner quadrant, upper inner quadrant, lower outer quadrant, and upper outer quadrant), and extraocular muscles (levator palpebrae superioris, superior rectus muscle, inferior rectus muscle, lateral rectus muscle, medial rectus muscle, superior oblique muscle, and inferior oblique muscle). The exclusion criteria were prior orbital surgery, orbital disease, or head and neck radiation therapy. 210 Arq Bras Oftalmol. 2021;84(3):209-13

### Randomization

Random software (random.org) was used to define a number between 1 and 100. The selected side was determined as left if the number was odd or as right if the number was even.

### Modified surgical technique of orbital exenteration

First, the incision line was marked around the orbit just inside the orbital rim. The dissection was then performed beneath the orbicularis oculi muscle to the orbital rim, and the periorbita was dissected from the orbital walls with a periosteal elevator. Enucleation scissors were passed around the orbital tissue to the apex, and any residual bleeding vessels were cauterized. At the end, the orbital cavity was packed with gauze sponges.

### Demographic data

Information was collected about the age and gender of all subjects.

### Histologic analysis

All specimens (lacrimal gland, optic nerve, fat tissue, and extraocular muscles) were fixed in a buffered 10% formaldehyde solution, embedded in paraffin, and processed for light microscopy at the Pathology Laboratory, Universidade Federal de Alagoas, Maceió, Brazil. For light microscopy, 4-µm-thick paraffin sections were stained with hematoxylin-eosin and periodic acid-Schiff.

### Immunohistochemical analysis

Immunohistochemical analysis was performed on 4-µm-thick paraffin sections using the peroxidase-labeled streptavidin-biotin method and an LSAB-Plus Kit according to the manufacturer’s instructions (Dako, Glostrup, Denmark). The paraffin sections were incubated with primary antibody dilutions, biotinylated link antibody, and peroxidase-conjugated streptavidin for 30 min each. The 3-chromogenic substrate 3-amino-9-ethyl carbazole was applied, and the samples were stained with Mayer’s hemalum.

A monoclonal antibody against the human lymphatic vascular endothelial-specific glycoprotein podoplanin D2-40 (mouse, 1:40; AbD Serotec) was used. Positive controls were performed on amygdala specimens, and negative controls were performed with control immunoglobulin G. Two investigators analyzed all paraffin sections.

### Detection of lymphatic vessels

The histologic criteria for a lymphatic vessel were thin-walled channels of endothelium without a welldeveloped basal membrane and with an erythro cytefree, irregular lumen. The immunohistochemical criteria were irregularly shaped, thin-walled vessels with an erythrocyte-free, irregular lumen and immunoposi tivity for podoplanin, D2-40.

### Ethical considerations

Informed consent was obtained for autopsy, orbital exenteration, histopathologic examination, and immunohistochemical analysis. The study was in compliance with the rules of the local research ethics committee and the tenets of the Declaration of Helsinki for experiments involving human tissues.

### Statistical analysis

Statistical analysis was performed using IBM SPSS Statistics software version 18.0 (SPSS, Chicago, IL, USA).

## RESULTS

### Demographic characteristics

The study included seven male and three female subjects, with four right and six left orbits. The mean age was 65.3 years, with a range of 33 to 102 years.

### Histologic analysis

According to the histologic criteria, the sections of lacrimal gland (palpebral and orbital lobes), optic nerve, fat tissue, and oculomotor muscles (levator palpebrae superioris, superior rectus muscle, inferior rectus muscle, lateral rectus muscle, medial rectus muscle, superior oblique muscle, and inferior oblique muscle) stained with hematoxylin-eosin and periodic acid-Schiff displayed thin-walled lymphatic vessels without a welldeveloped basal membrane and with an erythro cytefree, irregular lumen.

### Immunohistochemical analysis

**Figure 1:**
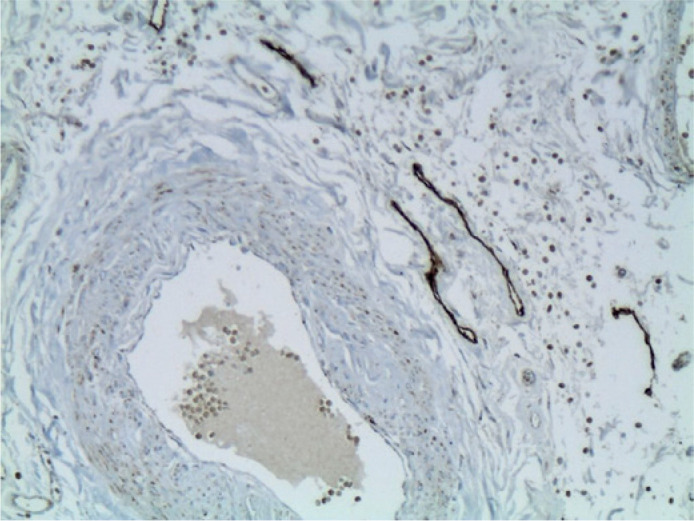
Immunohistochemical identification of lymphatic vessels reacting with podoplanin D2-40 in the lacrimal gland (original magnification x100).

**Figure 2:**
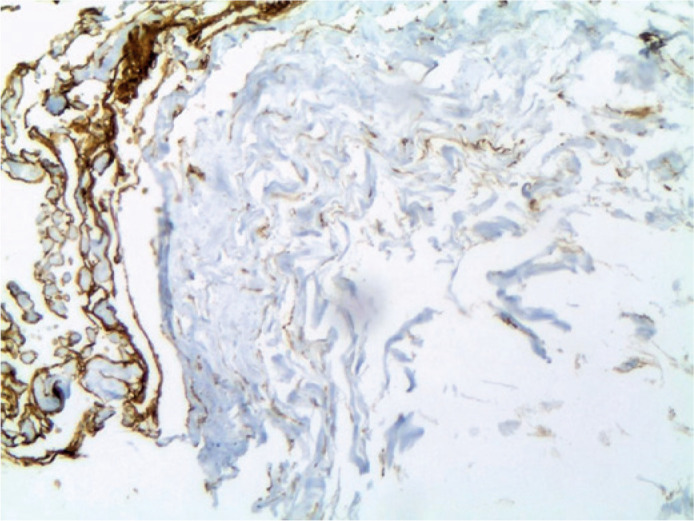
Immunohistochemical identification of lymphatic vessels reacting with podoplanin D2-40 in the dura mater of the optic nerve (original magnification x100).

**Figure 3:**
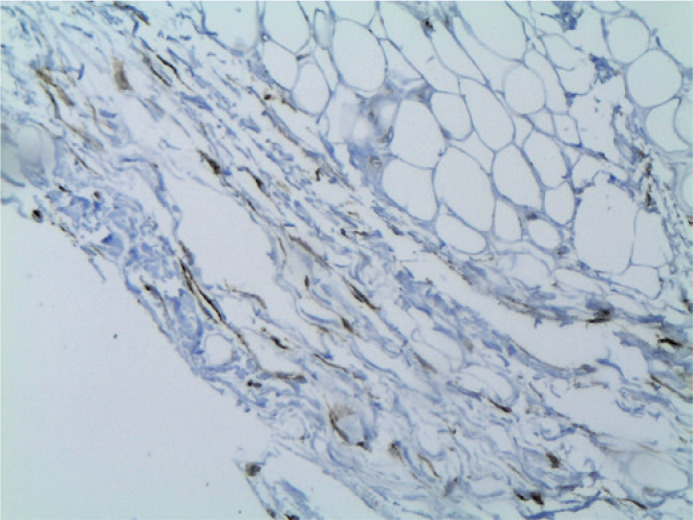
Immunohistochemical identification of lymphatic vessels reacting with podoplanin D2-40 in the orbital fat (original magnification x100).

**Figure 4:**
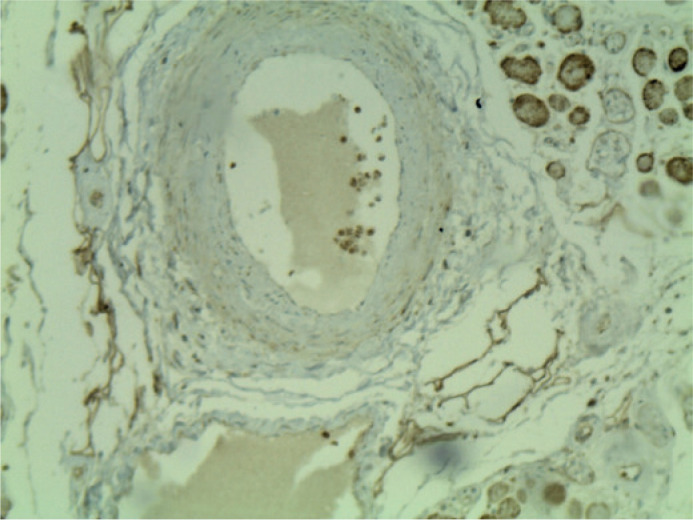
Immunohistochemical identification of lymphatic vessels reacting with podoplanin D2-40 in the extrisinc oculomotor muscle fascia and the connective tissue among the muscle fascicles (original magnification x100).

The sections of lacrimal gland (Figure 1), optic nerve (Figure 2), adipose tissue (Figure 3), and oculomotor muscles (Figure 4) stained positive for podoplanin D2-40.

## DISCUSSION

This study revealed lymphatic vessels in the human orbit, more precisely, in the lacrimal gland, optic nerve, fat tissue, and extraocular muscles, using light microscopy and immunohistochemical analysis with D2-40, a specific antibody for lymphatic vessels^(9)^.

### Lacrimal gland

The presence of lymphatic vessels in the human lacrimal gland was suggested by light microscopy in 1993^(7,10)^. The findings of the present study may contribute to a better understanding of the immune-mediated inflammatory diseases of the lacrimal gland and the spread of malignant neoplasms from and to the lacrimal glands^(5,6,11,12)^.

Some studies have indicated the role of the lymphatic vessels in the pathogenesis of inflammatory diseases, such as dry eye, primary Sjögren’s syndrome, and idiopathic dacryoadenitis. Lymphangiogenesis has been described in patients with dry eye and primary Sjögren’s syndrome^(11,12)^. A case series of idiopathic dacryoadenitis reported a possible pathogenic role of lymphoid neogenesis in lacrimal gland inflammation with lymphocytic aggregates^(13)^. Immunoglobulin G 4-related disease with involvement of the lacrimal glands, the mucosa associated-lymphoid tissues, and the lymph nodes is also characterized by lymphoid neogenesis with lymphocytic follicles^(5)^. Lymphatic vessels probably contribute to protecting the lacrimal glands against inflammation by providing a pathway for immune cells from the main lacrimal glands to the lymph nodes.

A research group has demonstrated the role of lymphangiogenesis in metastasis from the parotid gland and other regions^(14)^. These results may provide new therapeutic approaches to inhibiting metastasis^(15,16)^. Lymphatic neovascularization may be the main pathway for local and distant metastases of carcinomas and lymphomas from and to the lacrimal glands^(16)^. Chemotherapeutic agents may cause dry eye and epiphora as adverse effects, and therefore an adequate ophthalmological evaluation is mandatory during treatment with these agents.

### Optic nerve

Lymphatic vessels were observed by light microscopy, electron microscopy, and immunohistochemistry surrounding the optic nerve, more precisely, in the dura mater^(7,8)^. In humans, orbital lymphatic and blood vessels were first distinguished by morphologic criteria with specific 5’-Nase staining^(10)^. Some authors have reported the same finding with D2-40^(9,17)^, and others have provided information on the importance of this immunohistochemical antibody as a prognostic factor of invasion by tumors, including gliomas and meningiomas^(18-20)^, which are the most common optic nerve neoplasms.

### Fat tissue

Some authors have identified lymphatic vessels in orbital fat tissue from patients with idiopathic orbital inflammation and Graves’ orbitopathy by light microscopy and immunohistochemistry, respectively^(21,22)^. Lymphatic vessels have been described in all regions of the human body with adipose tissue except the orbital fat, which may be explained by the difficulty involved in the fixation of adipose tissue.

Benign orbital lesions, such as lymphangiomas, consist of abnormal lymphatic vessels and are frequently treated by surgical excision; however, most patients have multiple local recurrences^(23,24)^. Thus, new antilymphangiogenic therapies may be helpful in decreasing the local recurrence rates of these lesions.

Primary orbital malignant neoplasms are usually spread by lymphatic pathways. In addition, the human orbit may be affected by distant malignant tumors, such as lymphomas and melanomas^(25)^. Some authors have shown an association between tumor-related lymphangiogenesis and the increased risk of orbital invasion in squamous cell carcinoma and melanoma of the conjunctiva^(4,26)^. These findings may provide new therapeutic options for inhibiting metastases to and from the orbit^(2)^.

### Extraocular muscles

Lymphatic vessels have been detected by light microscopy and immunohistochemistry in the extrinsic oculomotor muscles in a primate model^(27)^. The current study revealed lymphatic vessels in the connective tissue of all the extrinsic oculomotor muscles. These findings corroborate the hypothesis of the lymphatic dissemination of nonocular melanoma^(28)^. Furthermore, some researchers have demonstrated intratumoral and peritumoral lymphatic vessels as prognostic factors for distant metastasis^(29,30)^.

The present study revealed lymphatic vessels in the human orbit, more precisely, in the lacrimal gland, optic nerve, adipose tissue, and extrinsic oculomotor muscles. These results may increase knowledge of orbital diseases, such as optic nerve tumors, idiopathic inflammation, Graves’ ophthalmopathy, lymphangioma, lymphoproliferative disease, and malignant epithelial neoplasms.
